# The role of PAF in immunopathology: From immediate hypersensitivity reactions to fungal defense

**DOI:** 10.1002/biof.1888

**Published:** 2022-09-29

**Authors:** Mariano Sánchez Crespo, Olimpio Montero, Nieves Fernandez

**Affiliations:** ^1^ Unidad de Excelencia Instituto de Biología y Genética Molecular, CSIC‐Universidad de Valladolid Valladolid Spain; ^2^ Departamento de Bioquímica y Biología Molecular, y Fisiología, Facultad de Medicina Universidad de Valladolid Valladolid Spain

**Keywords:** acetyl‐CoA, citrate, cytokines, dendritic cells, fungal infection, glucans, mitochondrial shuttles, phagocytes, platelet‐activating factor

## Abstract

Platelet‐activating factor (PAF, 1‐alkyl‐2‐acetyl‐*sn*‐glycero‐3‐phosphorylcholine) was discovered when the mechanisms involved in the deposition of immune complex in tissues were being scrutinized in the experimental model of rabbit serum sickness. The initial adscription of PAF to IgE‐dependent anaphylaxis was soon extended after disclosing its release from phagocytes stimulated by calcium mobilizing agents, formylated peptides, and phagocytosable particles. This explains why ongoing research in the field turned to the analysis of immune cell types and stimuli involved in PAF production with the purpose of establishing its role in pathology. This was spurred by the identification of the chemical structure of PAF and the enzymic mechanisms involved in its biosynthesis and degradation, which showed commonalities with those involved in eicosanoid production and the Lands' cycle of phospholipid fatty acid remodeling. The reassignment of PAF function in immunopathology is explained by the finding that the most robust mechanisms leading to PAF production are associated with opsonic and non‐opsonic phagocytosis, depending on the cell type. While polymorphonuclear leukocytes exhibit opsonic phagocytosis, monocyte‐derived dendritic cells show a marked preference for non‐opsonic phagocytosis associated with C‐type lectin receptors. This is particularly relevant to the defense against fungal invasion and explains why PAF exerts an autocrine feed‐forwarding mechanism required for the selective expression of some cytokines.

**Abbreviations**
AA: arachidonate
ACLY: ATP citrate lyase
ACSS: acyl‐CoA synthetase short‐chain family
ATF: activating transcription factor
COX: cyclooxygenase
cPLA_2_
: cytosolic phospholipase A_2_
CRE: cyclic AMP‐response element
FcγR: Fcγ receptor
HSCoA: reduced form of CoA
Ig: immunoglobulin
LPCAT: lysophospholipid acyltransferase
LPS: lipopolysaccharide
MAPK: mitogen‐activated protein kinases
LT: leukotriene
MDDCs: monocyte‐derived dendritic cells
NF‐κB: nuclear factor κB
PAF: platelet‐activating factor
PAFR: PAF receptor
PAMP: pathogen‐associated molecular pattern
PKC: protein kinase C
PG: prostaglandin
SYK: spleen tyrosine kinase
TCA: tricarboxylic acid
TLR: Toll‐like receptor
UPLC: ultra‐performance liquid chromatography/mass spectrometry
WT: wild type

## 1 THE INITIAL SETTING: BIOLOGICAL ACTIVITY AND CHEMICAL STRUCTURE

**FIGURE 1:**
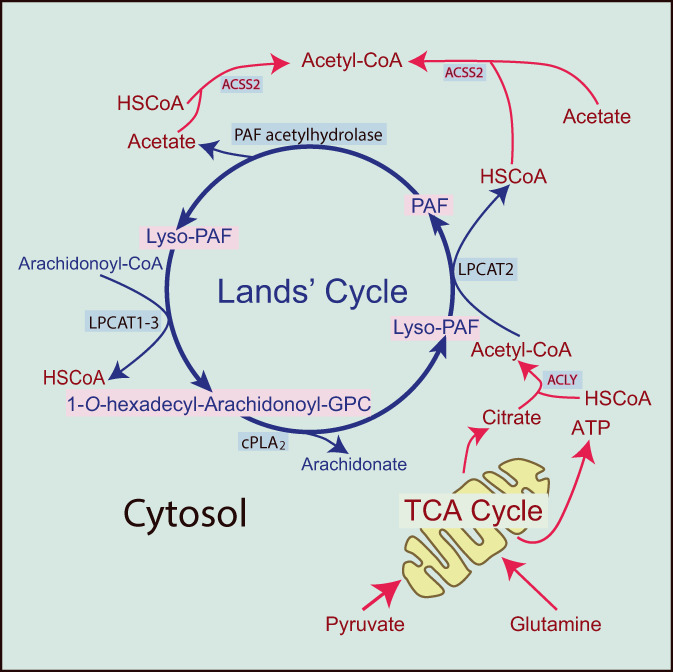
The connection of mitochondrial function with Lands' cycle. The Lands' cycle allows the conversion of structural phospholipids in internal bilayers into phospholipid mediators with a wide scope of biological actions. The compound 1‐*O*‐hexadecyl‐2‐arachidonoyl‐*sn*‐3‐glycerophorylphocholine is cleaved by cPLA_2_ into lyso‐PAF and AA. While AA can be converted into eicosanoids, lyso‐PAF can be acetylated by LPCAT2 to yield PAF. The reduced form of CoA (HSCoA) may be used by acyl‐CoA synthases and/or by ACSS2. The cycle continues by the deacetylation of PAF by PAF acetylhydrolase enzymes and reacylation of lyso‐PAF. Acetyl‐CoA can be produced in the nucleocytosolic compartment by ACLY or from HSCoA and acetate by ACSS2. Reprinted with permission from Ref. [35].

The existence of immunologic mechanisms driving the release of vasoactive mediators from rabbit platelets had previously been proposed, but the study by Benveniste, Henson, and Cochrane^1^ is the mainstay of the characterization of a soluble factor they dubbed platelet‐activating factor, from which the acronym PAF was derived. This study was in the wake of addressing the mechanisms implicated in the deposition of immune complexes in the acute serum sickness of rabbits, which involved a model of immunoglobulin (Ig) E‐mediated hypersensitivity that associated allergic reactions with tissue injury induced by the deposition of immune complexes in vessels. Acute serum sickness is produced by active immunization and occurs when large amounts of antigen reach the circulation. The archetypal example of this ailment was the administration of heterologous serum, which explains the use of the term serum sickness. As antibodies are produced in response to antigen load, immune complexes are formed, reach small vessels, and damage parenchymal tissues. Serum sickness stands as the most accurate model of damage caused by tissue deposition of immune complexes. Of note, a converse sequence of immune reactants giving rise to PAF production was observed some years later following intravenous administration of antibody to animals with high antigen load of *Cryptococcus neoformans*. This leads to intravascular formation of immune complexes that upon binding Fcγ receptors (FcγR) drive PAF production and the ensuing sequence of vascular leakage, circulatory shock, reduction in blood platelet count, and leukocyte margination, all of which comprising what is named acute lethal toxicity syndrome.^2^ These findings agreed with the previously reported effect of PAF on systemic circulation, which is characterized by a drop of blood pressure and massive plasma extravasation.^3^ As a corollary to these studies, myeloid cells emerged as the main producers of PAF, and this explains why PAF research focused on immune‐complex and leukocyte‐mediated tissue injury. This notion has recently been extended after disclosing that an IgG‐dependent and PAF‐mediated activation of myeloid cells may aggravate drug‐induced anaphylaxis by teaming up with the IgE pathway.^4^ In keeping with this view, a decreased serum level of the acetylhydrolase enzyme involved in PAF degradation (Figure 1) has been reported as a sensitive biomarker of severe anaphylaxis in children.^5^

A breakthrough in this field of research was the discovery of the chemical structure of PAF by Demopoulos, Pinckard, and Hanahan,^6^ who through enzymic and analytic approaches showed the identity between 1‐*O*‐alkyl‐2‐acetyl‐*sn*‐glycero‐3‐phosphorylcholine and PAF obtained from antigen‐sensitized rabbit basophils. In a further study starting from PAF activity obtained from rabbit basophils challenged *in vitro* to produce microgram quantities of naturally derived PAF, the same group successfully conducted a series of gas–liquid chromatographic and mass spectrometric analyses concluding that the naturally occurring PAF is the acetyl derivative of glyceryl ether phosphorylcholine.^7^ The chemical characterization of PAF prompted a series of studies on the routes involved in PAF biosynthesis and degradation, which unveiled the close association of PAF biosynthesis with phospholipase A_2_ hydrolysis of 1‐*O*‐alkyl‐2‐arachidonoyl‐*sn*‐3‐phosphorylcholine, the PAF precursor, and the acetylation of 1‐*O*‐alkyl‐2‐lyso‐*sn*‐3‐phosphorylcholine, thus mimicking the phospholipid deacylation and reacylation pathway called Lands' cycle (Figure 1). PAF biosynthesis depends on a first step catalyzed by intracellular cytosolic phospholipase A_2_ (cPLA_2_ group IVα), and a second reaction involving acetyl‐CoA:1‐*O*‐alkyl‐2‐lyso‐*sn*‐glycero‐3‐phosphorylcholine acetyltransferase.^8^ This enzyme was cloned in 2007 and assigned to the family of lysophospholipid acyltransferases with the acronym LPCAT2.^9^ It possesses a preferential ability to use acetyl‐CoA as the acyl donor, as compared with other LPCATs showing preference for long chain acyl‐CoAs. Both cPLA_2_ and LPCAT2 are Ca^2+^‐dependent enzymes and activated by phosphorylation. This explains their regulation by signals elicited by cell membrane receptors accessible to stimuli present in the extracellular milieu, for example, G‐protein‐coupled receptors, Fcγ and Fcε receptors, and the C‐type lectin receptor dectin‐1 involved in the recognition of β‐glucan, a pathogen‐associated molecular pattern (PAMP) contained in the external cell wall of fungi.

Conversely, given the limited effect of direct Ca^2+^‐driven signals associated with the bacterial lipopolysaccharide (LPS)/Toll‐like receptor (TLR) 4 system, the context of PAF in immune‐mediated tissue injury has focused on FcγR and FcεR mediated ailments and fungal infections, rather than on endotoxin shock. The field received further input after disclosing that PAF induces its pleiotropic effects through binding to a G‐protein‐coupled receptor (PAFR), which represented the first cloning of a receptor conveying signals elicited by a lipid mediator.^10^ Given that G‐protein‐coupled receptors signal via mitogen‐activated protein kinases (MAPK) and LPCAT2 is activated by phosphorylation at Ser‐34 by a cascade involving the p38 MAPK target MAPK‐activated protein kinase 2,^11^ a PAF cycle was posited where PAFR activation starts PAF biosynthesis. This mechanism was confirmed after scrutinizing the effect of different agonists. LPS induced phosphorylation of LPCAT2 within 30 min^10^ and increased LPCAT2 mRNA at 16 h.^9^ In stark contrast, PAFR activation by its cognate ligand induced PKCα‐mediated phosphorylation of LPCAT2 and PAF production within 30 s.^12^ This result gives support to the notion that PAF, by inducing its own production in a variety of settings,^13^ is an actual effector of tissue damage induced by environmental stressors.^14^ The dependence of PAF biosynthesis on cPLA_2_ explains why PAF biosynthesis parallels eicosanoid production and makes the mediators derived from phospholipid fatty acid remodeling a system whose activity precedes the transcriptional activation of genes involved in the inflammatory response, that is, the proinflammatory cytokines and cyclooxygenase (COX) 2. Of note, recent research unveiled that in addition to exerting proinflammatory effects on its own, PAF enhances the *trans*‐activation of some cytokine genes due to its ability to activate latent transcription factors via MAPKs.^15^

## 2 PAF PRODUCTION IN THE CONTEXT OF FUNGAL INFECTION

The initial report linking fungal patterns with PAF dates back to 1981, many years before the characterization of PAMPs and pattern‐recognition receptors as key components of the innate immune response, when Mencia‐Huerta and Benveniste^16^ showed a robust release of PAF from rat peritoneal macrophages phagocytozing zymosan particles and immune complexes. Zymosan contains the external cell wall of *Saccharomyces cerevisiae*, which is composed of layers of β‐glucans and α‐mannans and behaves like a fungal surrogate. The production of PAF was related to the insolubility of the particles and was characterized as an active process mediated by specific receptor/acceptor(s) for the phagocytozed particles present on macrophage membrane. These results were a turning point regarding the prevalent notions on the role of PAF in pathophysiology, because they opposed the translation of the IgE‐dependent mechanism found in rabbits to the rat‐mastocyte model.^17^ Fungi are engulfed by phagocytes through both opsonic and non‐opsonic phagocytosis. Opsonic phagocytosis depends on serum factors that interact with microbial products and in many cases drive activation of the complement system. This type of phagocytosis is particularly important in polymorphonuclears and this explains why opsonization of fungal particles is needed for PAF biosynthesis in these cells.^18^ This is due to the low expression of the functional isoform of the β‐glucan receptor dectin‐1 in polymorphonuclears,^19^ which makes it necessary the resort to the usage of α_m_β_2_ integrin, also known as complement receptor 3 (C3R), to recognize the C3bi portion of complement factor 3 to engulf and then inactivate fungal conidia. In contrast, cells of the monocytic/macrophagic lineage express functional isoforms of dectin‐1, the expression of which increases following differentiation into antigen‐presenting dendritic cells in the presence of GM‐CSF. This delineates two different settings regarding fungal defense. In the presence of polymorphonuclears, opsonic phagocytosis reaches central stage, while in clinical settings driving granulocytopenia and high risk of invasive mycosis, monocytic cells are the only available ones.

The study of the function of lipid mediators in the inflammatory response mainly focused on the early phase, while the late phase revolved around COX2‐produced prostaglandin (PG) E_2_. The seminal study by Harizi et al.^20^ showed that COX2‐produced PGE_2_ increased IL‐10 production, while decreasing IL‐12 p70 and driving down‐regulation of mouse dendritic cell function. A mechanistic insight was provided by the study of the role of CREB coactivators CREB‐binding protein (CBP) and CREB‐regulated transcriptional coactivator 2 (CRTC 2), also known as transducer of regulated CREB activity (TORC). In addition to the COX2‐dependent autocrine feed‐forward mechanism underpinning IL‐10 production, NF‐κB activation negatively regulated IL‐10 production, most likely due to a competition of both CREB and NF‐κB for a limited amount of CREB‐binding protein.^21^ These findings indicated that occupancy of G‐protein‐coupled receptors by lipid mediators paves the way for the transcriptional activation of cytokines and likely contribute to polarize the immune response to the Th17 type opposing fungal infection. The study by Suram et al.^22^ using *Candida* conidia and zymosan dissected the macrophage response to fungal patterns by showing that COX2 expression depended on the interaction with TLR2 and dectin‐1, while only dectin‐1 caused arachidonate (AA) release through cPLA_2_ activation dependent on the immunoreceptor tyrosine‐based activation motif‐like (ITAM) domain of dectin‐1 and the tyrosine kinase spleen tyrosine kinase (SYK). A study conducted in human monocyte‐derived dendritic cells (MDDCs) extended these results to cytokine expression and compared the responses with those elicited by engaging FcγRs, which also signal through SYK.^23^ This study prompted a more detailed analysis of the possible cooperation of lipid mediators on the expression of cytokines involved in the polarization of the immune response toward antifungal defense.

## 3 ROLE OF 5‐LIPOXYGENASE PRODUCTS AND PAF IN THE *TRANS*‐ACTIVATION OF *IL23A* GENE

The IL‐12 family of cytokines includes two heterodimers sharing the p40 chain encoded by the *IL12B* gene. IL‐12 p70 protein includes a p35 chain encoded by the gene *IL12A*, while IL‐23 contains a p19 chain encoded by the gene *IL23A*. The levels of those cytokines in the immune‐synapse microenvironment play a central role in the polarization of the immune response: IL‐12 p70 rules Th1 type response, while IL‐23 is involved in Th17 type polarization. This explains why the balance IL12 p70/IL‐23 is a critical factor driving anti‐fungal response. Several studies have shown a limited capacity of fungal patterns to induce IL‐12 p70, while the robust induction of *IL23A* mRNA expression explains the preferential production of IL‐23. This scenario makes it relevant the delineation of the mechanisms whereby *IL23A* gene is *trans*‐activated. The involvement of NF‐κB in the regulation of IL‐12 p70 and IL‐23 has convincingly been demonstrated, but this does not explain the predominant production of IL‐12 p70 in response to LPS, versus the association of fungal patterns with IL‐23, given the shared capacity of LPS and zymosan to activate the NF‐κB route. Recent studies directed to disclose the mechanisms underlying the transcriptional regulation of *IL23A* focused on the role of activating transcription factor (ATF) 2.^24^, ^25^, ^26^ ATF2 transcriptional activity depends on complementary phosphorylations on N‐terminal Thr‐69 by p38 MAPK and Thr‐71 by extracellular‐regulated kinases (MEK–ERK–MAPK), respectively.^27^ This mimics the regulation of PAF biosynthesis, where cPLA_2_ and LPCAT2 are regulated by MAPK‐dependent phosphorylation triggered by membrane receptors. Based on this notion, it was posited that lipid mediators acting on their receptors might yield signals through protein kinase C (PKC) and Ca^2+^ mobilization that synergizing with TLR2‐ and SYK‐dependent activation contribute to ATF2 phosphorylation and *IL23A trans*‐activation.

**FIGURE 2:**
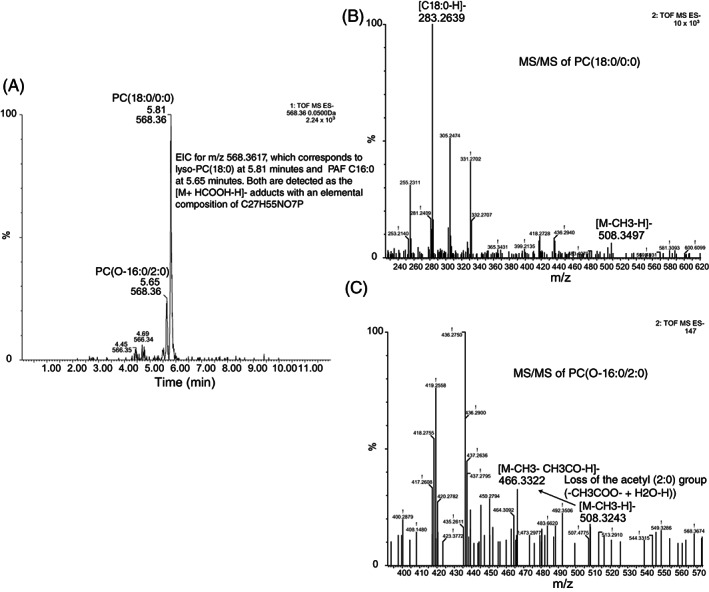
Human MDDCs produce PAF C16:0 in response to zymosan. (A) Extracted ion chromatogram (EIC) of *m/z* = 568.3617 for the formyl adducts [M + HCOOH‐H]^−^ of PC(18:0/0:0) and PC(O‐16:0/2:0) in the negative ion mode. (B) High energy mass spectrum corresponding to the peak of PC(18:0/0:0), where the peaks with *m/z* = 508.3497 and 283.2639 that correspond to the loss of a methyl group ([M‐CH_3_‐H]^−^) and to stearate, respectively, are shown. (C) High energy mass spectrum of PAF(O‐16:0/2:0) showing the *m/z* = 466.3322 that corresponds to the loss of the acetyl group as CH_2_=C=O after losing a methyl group (*m/z* = 508.3243).

**FIGURE 3:**
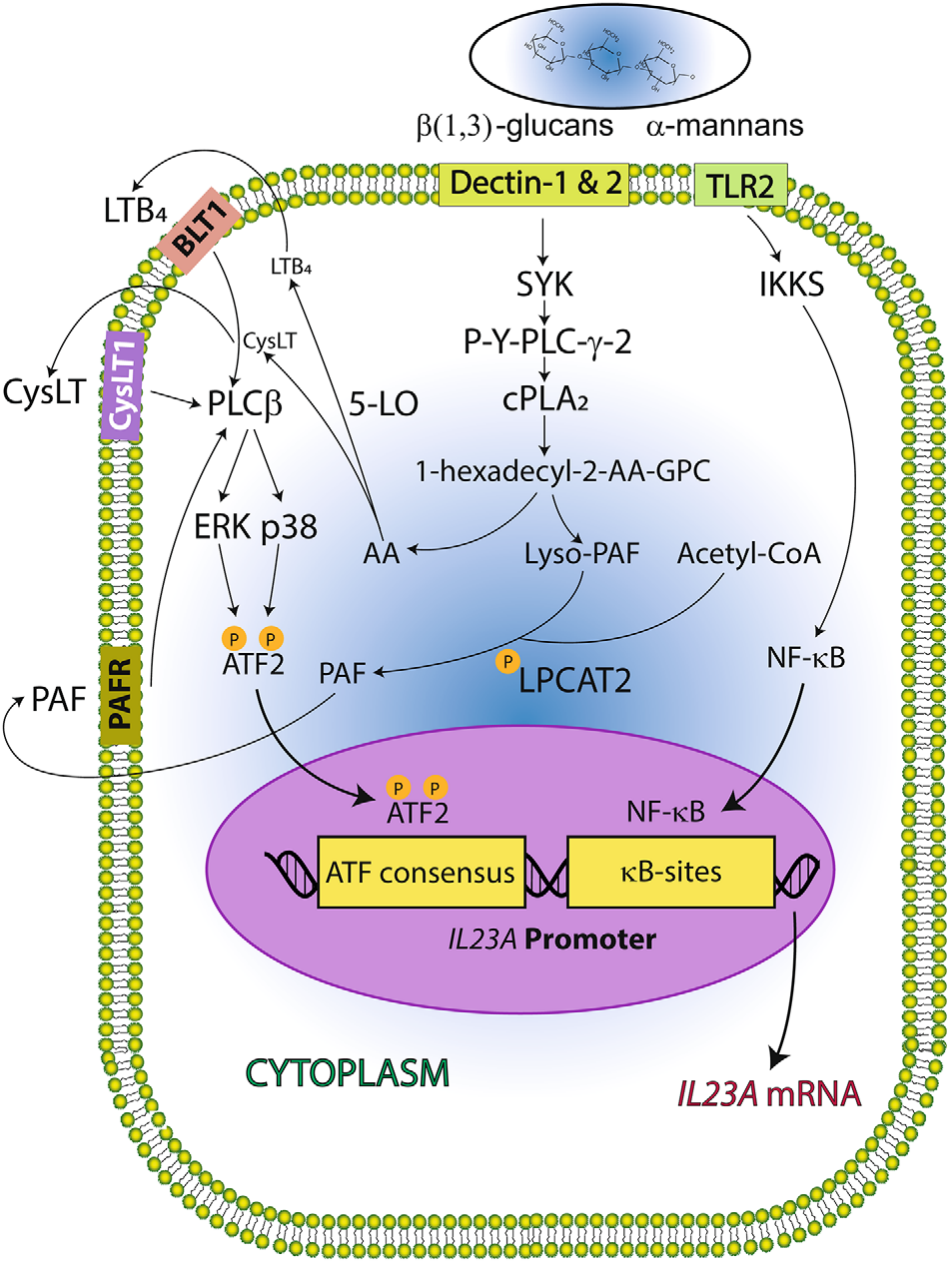
Lipid mediators and fungal patterns team up to trans‐activate *IL23A* gene. Binding of fungal patterns to the C‐type lectin receptors dectin‐1/2 activates spleen tyrosine kinase (SYK) and a signaling cascade that sequentially involves phospholipase C (PLC) γ2 activation, mobilization of Ca^2+^ and production of diacylglycerol, and cPLA_2_ activation. This yields arachidonate (AA) and lyso‐PAF from 1‐*O*‐hexadecyl‐2‐arachidonoyl‐*sn*‐3‐glycerophosphorylcholine, which by the activity of 5‐lipoxygenase (5‐LO) and LPCAT2, are converted into LT and PAF, respectively. These mediators are exported from the cytoplasm to the extracellular milieu where they can engage cell‐surface receptors to initiate a new signaling cascade through PLCβ, which reproduces some of the signaling steps induced by fungal patterns and contributes to the phosphorylation of ATF2. Binding of ATF2 and elements of the NF‐κB family to the *IL23A* promoter sets optimal conditions for *IL23* expression. BLT1, leukotriene 4 receptor 1; CysLT1, cysteinyl‐leukotriene receptor 1; PAFR, PAF receptor. Reprinted with permission from Ref. [15].

Zymosan induces a robust release of AA by MDDCs that may reach amounts as high as 1 μg/10^6^ MDDCs. This is followed by a significant production of leukotriene (LT) B_4_, PGE_2_, PGD_2_, and 12‐HETE. These eicosanoids decrease to prestimulation levels at 24 h, except for PGE_2_ and PGD_2_. LTE_4_ was detected at a concentration of ∼7 ng/10^6^ cells, which agrees with the reported concentrations at which its immediate precursor LTD_4_ elicits optimal stimulation of the CysLT1 receptor.^15^ Similar findings were observed in response to heat‐inactivated *Candida*, thus supporting the clinical relevance of the results to understand the pathophysiology of fungal infections. PAF C16:0 was detected upon stimulation of MDDCs with zymosan, while PAF C18:0 was found at negligible concentrations. PAF C:16 was unambiguously characterized after Bligh and Dyer extraction and ultra‐performance liquid chromatography–mass spectrometry (UPLC–MS). The different fragmentation pattern allowed distinguishing PAF C:16 from lysophosphatidylcholine PC(18:0/0:0), which share the same nominal mass and, hence, mass‐to‐charge ratio (Figure 2). The involvement of the LPCAT family of enzymes involved in fatty acid phospholipid remodeling was confirmed by the expression of mRNA transcripts of *LPCAT1* and *LPCAT2* at levels higher than *LPCAT3* mRNA, and an enzymic assay with acetyl‐CoA and lyso‐PAF in the cell‐free medium in the presence of both lyso‐PAF and acetyl‐CoA. When AA‐CoA was used as a co‐substrate, PAF formation was reduced.^15^ The capacity of MDDCs to respond to PAF was supported by the expression of the mRNA encoding *PAFR*. These results pointed at the involvement of LT and PAF in the transcriptional regulation of *IL23A* and agreed with the ability of these mediators to activate protein phosphorylation cascades. In keeping with this notion, MEK and p38 MAPK inhibitors blunted the Thr(P)‐69‐ATF2 binding to *IL23A* promoter elicited by zymosan in chromatin immunoprecipitation assays. Knockdown of *ATF2* mRNA with siRNA correlated with inhibition of *IL23A* mRNA, but it did not affect the expression of *IL12B* and *IL10* mRNA. These data indicated that zymosan‐induced *IL23A* mRNA expression relies on coordinated κB‐ and ATF2‐dependent transcription. Likewise, *IL23A* expression relies on complementary phosphorylation of ATF2 on Thr‐69 and Thr‐71 dependent on PKC and MAPK activities (Figure 3). The next notion guiding the study was addressing whether the lipid mediators produced following dectin‐1 engagement teamed up to enhance *IL23A trans*‐activation. Consistent with this view, the SYK inhibitor piceatannol, the cPLA_2_ inhibitor pyrrolidine‐1, and the 5‐lipoxygenase inhibitor zileuton induced a robust inhibition of P‐71Thr‐ATF2 binding to the *IL23A* promoter, which was paralleled by the inhibition of the expression of *IL23A* mRNA and further enhanced by the PAF antagonist WEB2086.^15^ Together, these results suggest that unlike LPS, zymosan elicits the generation of autocrine LTs and PAF, which contribute to the *trans*‐activation of *IL23A*. This yields IL‐23, the cytokine involved in maintaining the Th17 lineage,^28^ which in addition to its involvement in fungal defense, plays a role in the pathogenesis of inflammatory diseases such as psoriasis and intestinal inflammatory disease.

## 4 PAF AND THE BIOENERGETIC REWIRING ELICITED BY FUNGAL PATTERNS

**FIGURE 4:**
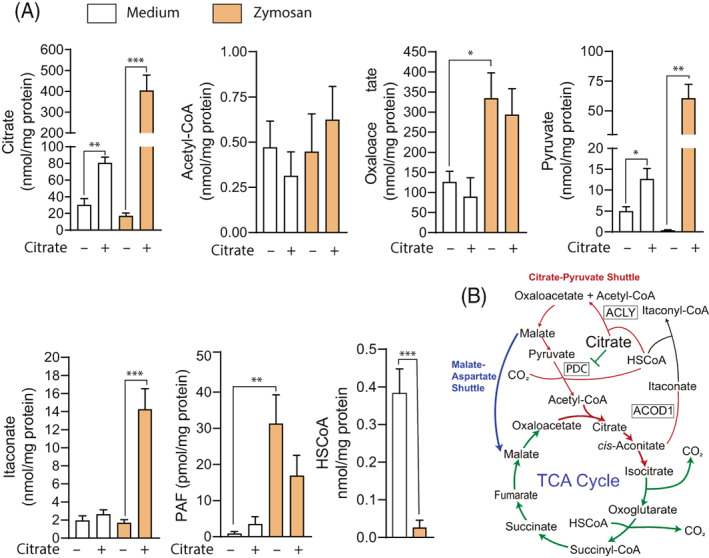
Effect of supplemental citrate on metabolic intermediates. (A) MDDCs were preincubated in the presence and absence of 5 mM citrate and then incubated in the presence of zymosan particles or medium. After 30 min of stimulation, MDDCs were washed to remove non‐incorporated citrate and intracellular metabolites extracted for UPLC‐MS analysis. Results represent mean ± SEM. *p < 0.05; **p < 0.01; ***p < 0.005. (B) Summary of the effects induced by exogenous citrate on TCA cycle intermediates. Red lines indicate steps showing increased metabolite levels. Metabolites involved in the mitochondrial shuttles are shown. ACLY, ATP citrate lyase; ACOD1, *cis*‐aconitate decarboxylase; PDC, pyruvate dehydrogenase complex. Reprinted with permission from Ref. [30].

The engagement of two enzymic reactions in PAF biosynthesis poses the question of which might be the limiting step. The cPLA_2_ reaction depends on binding of intracellular Ca^2+^ to an aminoterminal C2 domain, which drives translocation to the nuclear envelope and endoplasmic reticulum where substrate locates. Phosphorylation of Ser‐505 by MAPK enhances the enzyme activity and yields lyso‐PAF to LPCAT2, which finally incorporates acetate from acetyl‐CoA. Given the intense energetic rewiring caused by the phagocytosis of fungal patterns, this raises the question of how acetyl‐CoA disposal shapes PAF production. Acetyl‐CoA is a central metabolic intermediate used for acetylation of histones and metabolic regulators and in the synthesis of fatty acids, a process termed lipogenesis. Recent studies have disclosed that unlike LPS, fungal conidia and zymosan activate the energetic metabolism by simultaneously increasing glycolysis and oxidative phosphorylation.^29^, ^30^ This poses an immediate question given that acetyl‐CoA cannot be easily formed in the nucleocytosolic compartment, where it is widely utilized. The current model establishes that acetyl‐CoA is produced from the citrate exported from the mitochondria in exchange for malate by the SLC25A1 transporter. Nucleocytosolic citrate is converted into acetyl‐CoA and oxaloacetate by ATP citrate lyase (ACLY). A consequence of this break of the tricarboxylic acid cycle in monocytic cells is the need of other nutrients to maintain the downstream cycle. This is accomplished via glutaminolysis, which drives 2‐oxoglutarate in the mitochondrial matrix. Based on these notions, it was posited that the reported effect of citrate in fungal killing^31^ and cytokine expression^32^ could be due to its ability to generate nucleocytoplasmic acetyl‐CoA and then enhance the autocrine effect of PAF. Alternatively, citrate effect could depend on other actions, including the production of oxaloacetate and pyruvate from ACLY and cytoplasmic malic enzyme, respectively. Consistent with this view was the reduction of intracellular citrate by zymosan, and the ensuing notion that exogenous citrate can reverse this drop and through the production of nucleocytoplasmic acetyl‐CoA enhance PAF formation. Results showed that exogenous citrate increased the intracellular levels of citrate, pyruvate, and itaconate, but neither acetyl‐CoA nor PAF levels showed significant changes (Figure 4A). The ACLY inhibitor BMS303141 only reduced intracellular citrate in the presence of exogenous citrate. A cogent explanation for the steady levels of acetyl‐CoA could be the shortage of the reduced form of CoA (HSCoA) due to an increased consumption under the very demanding energetic conditions elicited by the phagocytic challenge (Figure 4B). Moreover, itaconate acts as a HSCoA trap due to the production of itaconyl‐CoA, which in turn, decreases the supply of succinyl‐CoA to the tricarboxylic acid (TCA) cycle from the α‐ketoglutarate dehydrogenase complex.^33^ An explanation for the apparent neutrality of acetyl‐CoA levels on PAF biosynthesis was suggested in early experiments directed to address the kinetics of the LPCAT2 reaction. Upon cell activation driving LPCAT2 phosphorylation, the reaction shows an increase of *V*_max_ mechanism (maximum velocity), whereas the *K*_*m*_ (Michaelis constant) for acetyl‐CoA is not affected.^18^, ^34^ This explains why PAF biosynthesis may be more dependent on lyso‐PAF production by cPLA_2_ than on citrate‐derived acetyl‐CoA and the marginal effect of supplemental citrate on zymosan‐induced PAF production (Figure 4A). These findings counter the view that the nucleocytoplasmic citrate/acetyl‐CoA systems exert its effects on cytokine expression by tuning the availability of acetyl‐CoA to LPCAT2 and indicate that the effect of citrate can be exerted through various mechanisms, that is, as a substrate, by product inhibition, by allosteric mechanisms, by supporting the citrate‐pyruvate shuttle, and by yielding itaconate.

Tellingly, in addition to the canonical formation of acetyl‐CoA from citrate, acetyl‐CoA can be produced from acetate and HSCoA by acyl‐CoA synthetase short‐chain family member (ACSS) 2. The involvement of these routes in the autocrine role of PAF production in cytokine formation was addressed in bone marrow‐derived dendritic cells (BMDCs) from mice with genetic deletion of the PAF receptor gene (*Ptafr*).^35^ While wild type (WT) mice showed a higher production of IL‐23 protein and *Il23a* mRNA than *Ptafr* ^*−/−*^ mice in response to zymosan, there was no difference in the response to LPS. Recent studies on the energetic rewiring induced by LPS disclosed that TNFα and IL‐1β induction needs the production of ATP in the mitochondrial electron transport chain from the NADH yielded by pyruvate dehydrogenase during pyruvate decarboxylation and the ensuing production of mitochondrial acetyl‐CoA.^36^ In keeping with this notion, blunting the production of pyruvate‐derived acetyl‐CoA with UK5099, an inhibitor of the mitochondrial pyruvate carrier, decreased IL‐23 production in WT mice in response to zymosan to an extent comparable to that observed in human MDDCs. Notably, acetate supplementation increased the production in WT mice and countered the effect of UK5099. Contrariwise, the effect of acetate was not observed in the *Ptafr* ^*−/−*^ mice.

These results indicate that a fraction of the role played by pyruvate in the production of cytokines can be associated with the autocrine production of PAF. The effect of supplemental citrate on cytokine production may involve several mechanisms, including the activation of mitochondrial shuttles. Consistent with the involvement of acetyl‐CoA in PAF biosynthesis, the production of IL‐23 induced by zymosan was acetate‐ and PAF‐dependent. The set of experiments involving inhibition of the mitochondrial pyruvate carrier and supplementation with citrate and acetate indicates that the supply of acetyl‐CoA to the Lands' route of PAF biosynthesis can be underpinned by both ACLY and ACSS2 activities (Figure 1).

## 5 CONCLUDING REMARKS

PAF was discovered in the context of cell–cell interactions involving basophils and platelets in a rabbit model involving IgE‐dependent reactions. This paved the way for a detailed analysis of immune cell types and stimuli involved in PAF production, aiming at establishing its role in immunopathology. Studies on polymorphonuclears, monocytic cells, and immediate hypersensitivity reactions reached central stage at that time. The identification of the phospholipid nature of PAF prompted biochemical studies disclosing commonalities with the mechanisms involved in eicosanoid production and the Lands' cycle of phospholipid fatty acid remodeling. The reassignment of PAF function in immunopathology took account of the outstanding production of PAF elicited by opsonic and non‐opsonic phagocytosis. The latter being particularly relevant in the context of fungal invasive infection, where PAF plays a major role in the autocrine feed‐forwarding mechanism involved in cytokine expression.

## FUNDING INFORMATION

The original work involved in this article has been supported by Valladolid Section of Asociación Española contra el Cáncer. Plan Nacional de Salud y Farmacia Grant SAF2017‐83079‐R and Grant PID2020‐113751RB‐I00 funded by MCIN/AEI/10.13039/501100011033. Junta de Castilla y León/Fondo Social Europeo Grants CSI035P17 and VA175P20. Proyecto SEAHORSE INFRARED: IR2020‐1‐UVA05. M.S.C. is part of the CSIC's Global Health Platform (PTI Salud Global).

## Data Availability

Data sharing not applicable. The review has been elaborated from our own published research.
